# Metabolomics of the tick-Borrelia interaction during the nymphal tick blood meal

**DOI:** 10.1038/srep44394

**Published:** 2017-03-13

**Authors:** J. Charles Hoxmeier, Amy C. Fleshman, Corey D. Broeckling, Jessica E. Prenni, Marc C. Dolan, Kenneth L. Gage, Lars Eisen

**Affiliations:** 1 Division of Vector-Borne Diseases, National Center for Emerging and Zoonotic Infectious Diseases, Centers for Disease Control and Prevention, Fort Collins, CO 80521, USA; 2 Proteomics and Metabolomics Facility, Colorado State University, Fort Collins, CO, 80523, USA.

## Abstract

The causal agents of Lyme disease in North America, *Borrelia burgdorferi* and *Borrelia mayonii*, are transmitted primarily by *Ixodes scapularis* ticks. Due to their limited metabolic capacity, spirochetes rely on the tick blood meal for nutrients and metabolic intermediates while residing in the tick vector, competing with the tick for nutrients in the blood meal. Metabolomics is an effective methodology to explore dynamics of spirochete survival and multiplication in tick vectors before transmission to a vertebrate host via tick saliva. Using gas chromatography coupled to mass spectrometry, we identified statistically significant differences in the metabolic profile among uninfected *I. scapularis* nymphal ticks, *B. burgdorferi*-infected nymphal ticks and *B. mayonii*-infected nymphal ticks by measuring metabolism every 24 hours over the course of their up to 96 hour blood meals. Specifically, differences in the abundance of purines, amino acids, carbohydrates, and fatty acids during the blood meal among the three groups of nymphal ticks suggest that *B. mayonii* and *B. burgdorferi* may have different metabolic capabilities, especially during later stages of nymphal feeding. Understanding mechanisms underlying variable metabolic requirements of different Lyme disease spirochetes within tick vectors could potentially aid development of novel methods to control spirochete transmission.

Lyme disease is the most commonly reported vector-borne disease in the United States, with more than 30,000 cases reported each year and indirect information sources indicating that the true numbers of annual Lyme disease cases are 10-fold higher^1^,^2^,^3^. Most of these cases occur in the Northeast and Upper Midwest, caused by the spirochete *Borrelia burgdorferi* and transmitted to humans primarily by the nymphal stage of the blacklegged tick, *Ixodes scapularis*^4^,^5^. In the Upper Midwest, the same tick also transmits another recently described Lyme disease spirochete, *Borrelia mayonii*^6^,^7^,^8^. In the continued absence of a human vaccine against these Lyme disease spirochetes, new approaches are needed to suppress vector ticks and disrupt the natural transmission of the spirochetes in their tick vectors and vertebrate reservoir hosts.

Various metabolomic methods, used to measure the abundance or flux of metabolites, or small molecule metabolites in a biological system, have been employed to provide insight into the complex molecular interactions that underpin the proliferation of vector-borne pathogens^9^,^10^,^11^,^12^,^13^, but similar efforts have been scarce for Lyme disease spirochetes^14^,^15^. A wide variety of instruments are employed for metabolomics projects, often utilizing a particular metabolite extraction solvent system^16^ followed by a chromatographic separation scheme (gas chromatography (GC), liquid chromatography (LC), hydrophilic interaction (HILIC)) coupled with mass spectrometry (MS)^17^. Non-targeted metabolomics studies provide global, unbiased coverage of metabolites^18^, closely reflecting the phenotype of an organism^19^. Analysis of metabolites involved in complex vector-pathogen interactions provides opportunities to discover innovative control methods for arthropod-borne pathogens^9^.

Pathogens that are maintained exclusively in arthropod vector-vertebrate reservoir host transmission chains often have considerably limited metabolic capacity, requiring exploitation of major vector/reservoir host cell functions for the acquisition of metabolic intermediates for development^10^,^20^. This is the case for *B. burgdorferi*, which in the eastern United States is maintained in a transmission chain involving *Ixodes* ticks, particularly *I. scapularis*, and various vertebrate reservoir hosts^21^, and presumably also for *B. mayonii*. Because *B. burgdorferi* spirochetes lack pathways for *de novo* biosynthesis of nucleotides, amino acids, fatty acids, and enzyme cofactors, they depend on the blood meal for metabolic intermediates and for intra-tick migration signals when residing within the tick vector^15^,^22^,^23^. Knowledge of *Borrelia* metabolism within the tick and the metabolic impact of *Borrelia* colonization of *I. scapularis* is limited. However, a metabolic cascade in the tick gut is initiated upon ingestion of blood, which also affects the phenotype of the colonizing pathogen^24^,^25^. Within unfed nymphs, nutrient deprived spirochetes remain in a metabolic state that has been largely uncharacterized^24^,^26^. The number of spirochetes within the tick gut remain at a low level during the first 24 hours of nymphal feeding^27^,^28^, but increases exponentially as the feeding progresses^29^, placing an increased metabolic demand on the tick.

By studying metabolic processes of *Borrelia*-infected ticks, in our case *B. burgdorferi* and *B. mayonii*, we can gain a more detailed understanding of intricate vector-pathogen interactions as well as explore variation in metabolic requirements among different Lyme disease spirochetes as they are exposed in the tick midgut to blood ingested from a vertebrate blood meal host, start to multiply and penetrate the tick midgut to reach the salivary glands, and are transmitted to the vertebrate tick host. Such knowledge can facilitate discovery of metabolites with potential utility for novel methods to control the spirochetes within the vector tick. While the roles of specific metabolites have previously been investigated^21^,^30^,^31^,^32^,^33^,^34^, this is the first account of the global metabolome of *Borrelia*-infected ticks during the period of nymphal blood feeding and spirochete transmission. Though we were able to make parallels to metabolites in other arthropod vector-pathogen interactions, additional research is necessary to investigate the impact of specific metabolites on the ability of *Borrelia* spirochetes to proliferate within and be transmitted by tick vectors.

## Methods

### Blood feeding by nymphal ticks

The ticks used in this study came from a colony maintained at The Centers for Disease Control and Prevention-Fort Collins, and originated from adults collected in multiple locations in Fairfield County, Connecticut in the fall of 2013. Nymphal ticks used in the experiments included uninfected *I. scapularis* nymphs and nymphs infected, by feeding on infectious mice in the preceding larval stage, with either *Borrelia burgdorferi* strain B31^35^ or the *Borrelia mayonii* type strain (MN14–1420)^36^. Nymphal blood meals were taken from 8–12 week old female *Mus musculus* CD-1 outbred mice (Charles River Laboratories, Wilmington, Massachusetts, USA). At the time of feeding, the nymphs were approximately 10 weeks post-molt.

Nymphs (n = 50) from each of the three experimental groups (*B. burgdorferi*-infected, *B. mayonii*-infected, and uninfected control) were collected before commencement of feeding to represent pre-feed sample specimens. Individual mice were exposed to feeding by 20 nymphs, allowed to select their feeding sites after being brushed onto anesthetized mice, from one of the three experimental groups, for a total of 9 mice infested by *B. burgdorferi*-infected nymphs, 12 mice by *B. mayonii*-infected nymphs and 8 mice by uninfected nymphs. Mouse numbers were determined based upon the infection rate of ticks, in order to generate the required numbers of infected nymphs per time point. Subsets of these mice were exposed to nymphal feeding for 24 hours, 48 hours, 72 hours and 96 hours, with the last time point representing complete feeding. At the assigned time point, all nymphs were removed from the mice or recovered from a water surface over which the mice were held to allow for collection for fed and detached ticks. No difference in the total blood meal volume (via tick weight) of replete ticks was detected (data not shown).

### Processing of nymphs for analysis of metabolites

After being collected, ticks were quenched in ice-cold 100% HPLC-grade methanol (Sigma Aldrich, St. Louis, MO) and stored at −80 C. For processing, individual ticks were separated and added to tubes containing 100 μl of cold 100% methanol. Approximately 10–2.3 mm chrome steel beads (Biospec Products Inc., Bartlesville, OK) were added and the sample was homogenized using a Mini-beadbeater (Biospec Products Inc.) for 1 minute.

After bead beating, the samples were centrifuged at 10,000 xg for 1 minute. The supernatant was removed into a clean tube. To wash the beads, 100 μl of fresh 100% methanol was added to the tube containing the beads, briefly vortexed, and centrifuged at similar conditions. The supernatant was removed and added to the previously collected sample to produce a final volume of 150 μl of methanol. All samples were placed in a rotary evaporator and evaporated with vacuum until a sample volume of 50 μl was reached.

After homogenization, the remaining pellet was subjected to PCR to determine the infection status of individual ticks using combined detection of *I. scapularis* actin, as a control for both the DNA purification and the PCR testing, and the spirochete flagellar filament cap (*fliD*) target, which is present in both *B. burgdorferi* and *B. mayonii*^6^,^37^,^38^,^39^. A modified multiplex TaqMan PCR assay was used as described^37^,^38^,^39^. Any remaining methanol was evaporated from the pellet and the pellet resuspended in 50 μl DNase-free water. The pellet suspension was boiled at 95 °C for 15 minutes and briefly centrifuged to remove large debris. 4.8 μl of the pellet supernatant was removed to an Axygen 96 well plate (BioSpec Products, Inc.) to which 5.2 μl of master mix containing iQ Multiplex Powermix (Bio-Rad, Hercules, CA, USA), and primers in a final concentration of 300 nM and probes in a final concentration of 200 nM. The PCR cycling conditions were 95 °C for 3 minutes to denature DNA followed by 40 cycles of 95 °C for 10 seconds and 60 °C for 1 minute on a C1000 Touch thermal cycler with a CFX96 real time system (Bio-Rad). After determining the infection status of individual ticks, the metabolite-containing methanol extraction collected previously was combined to generate a pool of metabolites from 10 infected ticks per time point (pre-feed and after 24 hours, 48 hours, 72 hours or 96 hours of feeding) and infected experimental group (*B. burgdorferi*-infected or *B. mayonii*-infected). Uninfected control ticks fed concurrently alongside the infected-tick feed, generating 3 pools of 10 uninfected control ticks per time point. Uninfected control ticks were processed and tested individually via PCR as described above to confirm their uninfected status.

### GC-MS Analysis

Metabolite extracts were dried under nitrogen, re-suspended in 50 μL of pyridine containing 15 mg/mL of methoxyamine hydrochloride, incubated at 60 °C for 45 minutes, sonicated for 10 minutes, and incubated for an additional 45 minutes at 60 °C. Next, 25 μL of N-methyl-N-trimethylsilyltrifluoroacetamide with 1% trimethylchlorosilane (MSTFA + 1% TMCS, Thermo Scientific, Waltham, MA) was added and samples were incubated at 60 °C for 30 minutes, centrifuged at 3000 xg for 5 minutes, cooled to room temperature, and 80 μL of the supernatant was transferred to a 150 μL glass insert in a GC-MS autosampler vial. Metabolites were detected using a Trace GC Ultra coupled to a Thermo ISQ mass spectrometer (Thermo Scientific). Samples were injected in a 1:10 split ratio twice in discrete randomized blocks. Separation was achieved using a 30 m TG-5MS column (Thermo Scientific, 0.25 mm i.d., 0.25 μm film thickness) with a 1.2 mL/minute helium gas flow rate, and the program consisted of 80 °C for 30 seconds, a ramp of 15 °C per minute to 330 °C, and an 8 minute hold. Masses between 50–650 m/z were scanned at 5 scans/second using electron impact ionization. The ionization source was cleaned and retuned and the injection liner replaced between injection replicates.

### Data Analysis and Statistics

For each sample, raw data files were converted to.cdf format, and a matrix of molecular features as defined by retention time and mass (m/z) was generated using XCMS software in R^40^ for feature detection and alignment. Raw peak areas were normalized to total ion signal in R, outlier injections were detected based on total signal and PC1 of principle component analysis, and the mean area of the chromatographic peak was calculated among replicate injections (n = 2). Due to normalization of the dataset, the quantitation of each molecular feature is described using Relative Abundance (RA). Features were grouped using RAMClustR^41^, which groups features into spectra based coelution and covariance across the full dataset, whereby spectra are used to determine the identity of observed compounds in the experiment. Compounds were annotated based on spectral matching to NISTv14 and GOLM spectral databases, using the RAMSearch program^42^. GOLM retention index was plotted against retention time to provide additional retention based confidence to annotations. Annotation confidence is reported as defined by the Metabolomics Standards Initiative^43^, with the majority of annotations considered level 2, and a few level 3 (chemical class) based annotations. The peak areas for each feature in a spectrum were condensed via the weighted mean of all features in a spectrum into a single value for each compound. Analysis of variance was conducted on each compound using the aov function in R, and p-values were adjusted for false positives using the Bonferroni-Hochberg method in the p.adjust function in R^44^. PCA was conducted on mean-centered and pareto variance-scaled data using the pcaMethods package in R. The precision of the analytical method is described by the closeness of repeated individual measures of quality control (QC) samples. Using the coefficient of variation (CV) as a measure of precision in our dataset, 80% of the compounds in the dataset demonstrated coefficient of variation (CV) values for QC samples of less than 15.3%, indicating a high degree of precision (Supplementary Figure 6)^45^.

### Regulatory compliance

Animal use and experimental procedures were in accordance with an approved protocol on file with the Centers for Disease Control and Prevention Division of Vector-Borne Diseases Animal Care and Use Committee.

## Results and Discussion

In this study, we describe the metabolic phenotypes associated with infection by the Lyme disease spirochetes *B. burgdorferi* and *B. mayonii* in *I. scapularis* nymphal ticks. All metabolites discussed herein are significant by ANOVA by both treatment and time. In our dataset, 480 of 567 (85%) compounds in the measured or analyzed metabolome were significantly different, typical of many untargeted metabolomics studies. We were able to annotate 114 of 567 (20%) metabolites. The statistically significant metabolites detected are reported in Table 1.

### Purine metabolism

Purine-based nucleotides, present in all living organisms, are fundamental components of many crucial biomolecules such as DNA, RNA, ATP, and coenzymes^46^. For many bacterial pathogens, purine metabolism is required for growth and virulence^47^,^48^,^49^. While mammals can synthesize purines *de novo*, many bacterial pathogens lack the necessary molecular machinery and must salvage purines from their host. The role of purine salvage has been well recognized in *Salmonella typhimurium* during human infections^50^ and for a variety of vector-borne protozoa and helminths^46^.

The genome of *B. burgdorferi* does not contain the genes encoding the enzymes required for *de novo* purine synthesis nor for the classical salvage pathway^47^. *B. burgdorferi* obtains both purine bases and deoxynucleotides via novel purine salvage pathways, directly from the host while residing in a vertebrate reservoir, or from tick-ingested vertebrate blood while residing within the tick vector^34^,^47^,^48^. The most abundant purine in mammalian blood is hypoxanthine^51^. The specific purine salvage abilities of *B. mayonii* are yet-undetermined.

**Figure 1:**
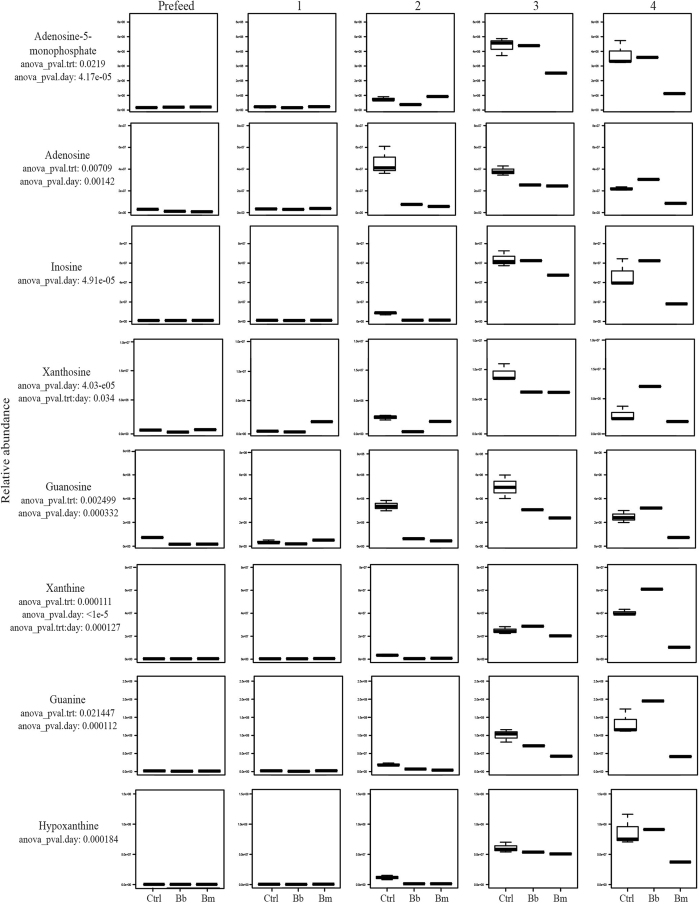
Purine Pathways. Accumulation over time of statistically significant (ANOVA p ≤ 0.05) metabolites involved in purine metabolic pathway in uninfected (control) *Ixodes scapularis* nymphs versus nymphs infected with the Lyme disease spirochetes *Borrelia burgdorferi or Borrelia mayonii*.

Many compounds involved in purine metabolism were statistically significant by ANOVA by both treatment and time in our dataset (Table 1). These data provide evidence that, during colonization and replication of *B. burgdorferi* and *B. mayonii* in feeding *I. scapularis* ticks, these spirochetes exert a significant demand on purines from the incoming vertebrate blood meal compared to the uninfected control ticks (Fig. 1, Supplementary Figure 1). Additionally, the data indicate possible differences in purine metabolism between the two species (Fig. 1, Supplementary Figure 1). Because *Borrelia* are able to sequester metabolites from the vector and the vertebrate host blood meal in lieu of *de novo* synthesis, the spirochetes have lost many genes encoding biosynthetic machinery. While incidences of gene loss from more metabolically competent ancestors have been previously identified in *B. burgdorferi* and other pathogenic host-associated bacteria^22^,^52^, additional research is required to evaluate the full metabolic capacity of *B. mayonii*.

### Amino Acids

A wide variety of arthropod-borne pathogens place significant metabolic demands on their vectors. The dependence on the vector, and especially the vertebrate blood meal it ingests, is evidenced by the lack of *de novo* synthesis capabilities of amino acids. In the vertebrate host, *Plasmodium falciparum* malaria, as well as *Leptospira* and *Treponema* spirochetes, harvest essential amino acids from plasma and cell hemoglobin^53^,^54^. In the sand fly vector, *Leishmania major* utilizes amino acids from the ingested blood meal as an energy source for glycolysis and related metabolic cycles^55^, while the plague bacterium *Yersinia pestis* catabolizes amino acids from the flea vector blood meal as a primary carbon source during colonization of the flea gut^56^.

*B. burgdorferi* also lacks the capacity for the de novo synthesis of amino acids and must sequester them from the vertebrate host or from the blood meal ingested by the tick vector^22^. Isoleucine, leucine, lysine, serine, and asparagine are the most common amino acids within the *B. burgdorferi* genome sequence^22^, but very little is known about how they are acquired within *I. scapularis.* In our dataset, the aliphatic and hydrophobic amino acid classes demonstrate the most significant differential abundance among treatment groups (Table 1, Supplementary Figure 2).

Interestingly, all statistically significant amino acids for both *B. burgdorferi* and *B. mayonii* display the same general trend (Supplementary Figure 2). In most cases, amino acid abundance in tick groups infected with *B. burgdorferi* remains at a lower level than uninfected controls for the duration of the blood meal (Supplementary Figures 2 and 3). Amino acid abundance in tick groups infected with *B. mayonii* follows the same trend, but sharply declines for each amino acid around day 3 (Supplementary Figure 2). Amino acid abundance in both uninfected tick groups and *B. burgdorferi*-infected tick groups follow the same general trend for most amino acids, with the exception of differences in the utilization of glutamine and urea (Supplementary Figure 2H,Q). Since the spirochetes must harvest amino acids from the tick’s blood meal, it seems appropriate that the abundance of amino acids would continue to decrease as feeding continues. Overall, our data show a difference in the impacts of *B. mayonii* and *B. burgdorferi* on the tick amino acid metabolism (Supplementary Figures 2 and 3).

### Carbohydrates

*B. burgdorferi* can utilize a finite number of carbohydrates as energy sources, including glucose, maltose, glycerol, mannose, trehalose, chitobiose, and N-acetylglucosamine (GlcNAc)^30^,^57^,^58^. The presence of three putative phosphotransferase-type glucose transporter genes in the genome of *B. burgdorferi* suggests that glucose is likely a primary energy source^30^,^59^. This is especially true for spirochetes within vertebrate reservoir hosts and when colonizing *I. scapularis* after a blood meal, as glucose is the most prevalent carbohydrate and most efficient energy source in mammalian blood^33^,^60^,^61^. During the period prior to nymphal feeding, when glucose is scarce, *B. burgdorferi* relies on glycerol and a finite supply of chitobiose to fuel glycolysis^33^. Furthermore, glycerol is required by *Borrelia* for maximum fitness during the tick phase of the enzootic cycle and subsequent transmission^33^. Maltose is not required by *B. burgdorferi* during mammalian infection^57^. However, the *B. burgdorferi* genome encodes several phosphoenolpyruvate:phosphotransferase (PTS) systems used to acquire maltose, suggesting that maltose may also be used in glycolysis^22^,^59^. Although previous studies hypothesized that *Borrelia* could utilize galactose as an energy source^22^, no evidence has been found to support this hypothesis^30^.

**Figure 2:**
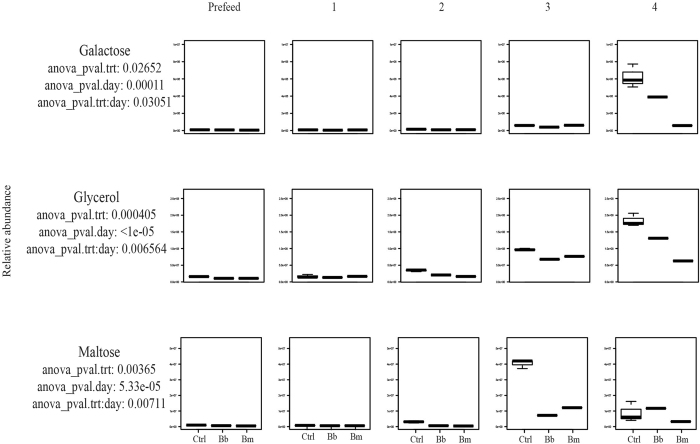
Carbohydrates. Accumulation over time of statistically significant (ANOVA p ≤ 0.05) carbohydrates in uninfected (control) *Ixodes scapularis* nymphs versus nymphs infected with the Lyme disease spirochetes *Borrelia burgdorferi or Borrelia mayonii*.

In our study, galactose, glycerol, and maltose are statistically significant by both treatment and time (Table 1). Our data show that all three *I. scapularis* groups utilize all detectable galactose until day 3, when the relative abundance begins to increase sharply in *B. burgdorferi*-infected tick groups (Fig. 2, Supplementary Figure 4). By day 4 of feeding, in tick groups infected with *B. burgdorferi*, the abundance of galactose decreases more so than within uninfected control tick groups, but less than *B. mayonii*-infected tick groups, which use significantly more galactose compared to the other groups (Fig. 2, Supplementary Figure 4). These data suggest that galactose may be an important carbohydrate involved in the development of spirochetes within the tick, represented by the lower relative abundance of galactose in tick groups infected with *B. burgdorferi* or *B. mayonii* compared to uninfected control ticks (Fig. 2, Supplementary Figure 4). Our data also suggest that *B. mayonii* and *B. burgdorferi* may differ in their metabolic capacities within vector ticks during the later stages of infection, especially with regard to specific carbohydrate utilization and availability (Fig. 2, Supplementary Figure 4).

Within *I. scapularis*, glycerol is involved in glycerophospholipid metabolism (KEGG map00564), glycerolipid metabolism (KEGG map00561), and ether lipid metabolism (KEGG map00565). While there is a low abundance of glycerol present in the early stages of feeding glycerol availability begins to spike upward at day 2 of feeding (Fig. 2, Supplementary Figure 4). As the blood meal arrives and is processed within the tick, ATP input is required to convert glycerol into a more usable form: glycerol-3-phosphate (also significant by treatment, time, and treatment:time) (Table 1). Glycerol-3-phosphate can then be converted into dihydroxyacetone phosphate by glycerol-3-phosphate dehydrogenase, using energy cofactors NADH and NAD+.

In both *I. scapularis* and in *B. burgdorferi*, maltose is involved with starch and sucrose metabolism, phosphotransferase systems, and ABC transporters (KEGG C00208). Our data show that the abundance of maltose in the uninfected control tick groups and the *Borrelia*-infected tick groups is similar early in the feeding process, but differs vastly 3 days after attachment (Fig. 2, Supplementary Figure 4). Post attachment (day 3), a significantly higher abundance of maltose is present in uninfected tick groups than *Borrelia*-infected tick groups, falling to a comparable abundance in all groups by day 4 (Fig. 2, Supplementary Figure 4). Because maltose accumulates more rapidly in the uninfected tick groups than in the infected tick groups, *B. burgdorferi-*infected tick groups likely have a greater demand for maltose from the tick early on in feeding. *B. mayonii* follows the same general trend as the uninfected control albeit with a lower relative abundance, suggesting that *B. mayonii*-infected tick groups and *B. burgdorferi*-infected tick groups may differ slightly in their maltose requirements during feeding (Fig. 2, Supplementary Figure 4). Maltose may be used as a carbon source until a certain threshold, when it may become involved in metabolite shuttling, as has been demonstrated in *Borrelia* phosphoenolpyruvate phosphotransferase systems (PEP-PTS)^59^,^61^, and in other bacteria^57^.

### Fatty acids

Fatty acids are a major component of phospholipid bilayers in cell membranes and play important roles as energy sources for many organisms including bacteria^62^. Some bacteria, such as *Coxiellia burnetii*, and *Chlamydia* spp. acquire lipids from their vertebrate host to establish and maintain infections^63^,^64^. Within bacterial and mammalian cell membranes and phospholipids, phosphatidylethanolamine is readily available^65^ to be broken down into ethanolamine and glycerol for use in glycerophospholipid metabolism, glycosylphosphatidylinositol-anchor biosynthesis, and pathogenesis^66^. Notably, ethanolamine is a critical metabolite used by enteric bacteria including *S. typhimurium*^67^, *Escherichia coli*^68^, EHEC (*E. coli* O157:H7)^69^, and *Enterococcus faecalis*^70^, in pathogenesis 69,71,72,73,74,75 and for energy^71^.

Because *B. burgdorferi* are not able to synthesize fatty acids or cholesterol, elongate fatty acid chains, nor beta-oxidize exogenous fatty acids *de* novo^76^,^77^, they rely heavily on their vertebrate hosts or the tick blood meal for these compounds^22^. Outer membranes of *B. burgdorferi* contain phosphatidylcholine, phosphatidylglycerol, and lipoproteins, but are mainly composed of cholesterol, which is processed to make cholesterol-glycolipids^77^,^78^,^79^,^80^,^81^. Free cholesterol and cholesterol-glycolipids within *B. burgdorferi* likely play important roles in the mediation of tick-cell interactions and create opportunities for lipid-lipid interactions with eukaryotic-like lipid rafts^78^,^82^. Lipid rafts may have implications during the enzootic cycle^83^.

**Figure 3:**
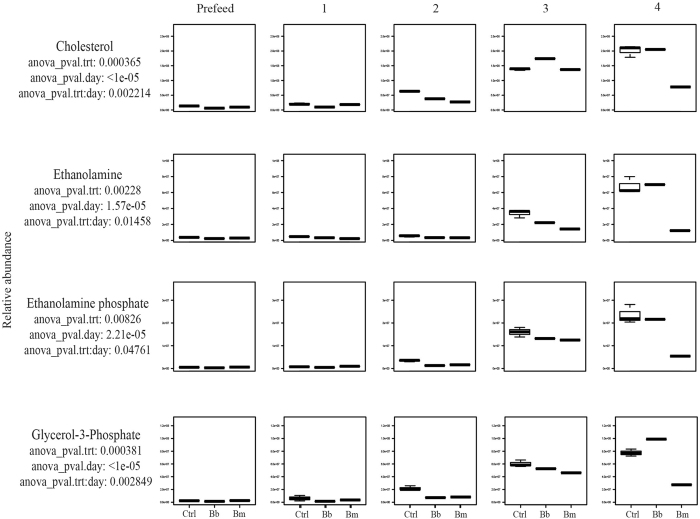
Fatty Acids. Accumulation over time of statistically significant (ANOVA p ≤ 0.05) lipids in uninfected (control) *Ixodes scapularis* nymphs versus nymphs infected with the Lyme disease spirochetes *Borrelia burgdorferi or Borrelia mayonii*.

In our dataset, total cholesterol abundance is low in all 3 tick groups at days 0 and 1 (Fig. 3, Supplementary Figure 5). After day 1, cholesterol begins to accumulate in the uninfected tick groups while *B. burgdorferi* and *B. mayonii*-infected tick groups demonstrate a lower abundance, potentially indicating increased acquisition of cholesterol by the spirochetes (Fig. 3, Supplementary Figure 5). By day 3, the cholesterol abundance in *B. burgdorferi-*infected tick groups rises above both *B. mayonii-*infected tick groups and control tick groups (Fig. 3, Supplementary Figure 5). The trend over time in *B. burgdorferi*-infected tick groups may reflect incoming cholesterol being used for tick membrane remodeling and for metabolic energy in the beginning of the feeding cycle. Since free cholesterol has been shown to be required for *B. burgdorferi*-eukaryotic cell interactions via lipid rafts^78^, this could potentially explain why cholesterol increases in abundance towards the end of the feeding cycle.

Also statistically significant (ANOVA p ≤ 0.05 by treatment, time, and treatment: time) in our dataset are ethanolamine phosphate, ethanolamine, and, as mentioned previously, glycerol-3-phosphate (Fig. 3, Supplementary Figure 5), all of which are involved in glycerophospholipid metabolism (KEGG map00564). These metabolites follow the same general trend in all 3 tick groups during days 0–3. The abundance of ethanolamine phosphate, ethanolamine, and glycerol-3-phosphate are low during days 0–2, but by day 3 post attachment, the abundance of each metabolite sharply increases in all three tick groups, followed by a rapid decline in only the *B. mayonii*-infected tick groups (Fig. 3, Supplementary Figure 5). Similar to the trend of cholesterol abundance over time, the abundance of ethanolamine, ethanolamine phosphate, and glycerol-3-phosphate during feeding may be described by the changing roles of these particular metabolites. At the start of the blood meal, exogenous carbon sources are necessary to synthesize new membrane components; as the tick reaches repletion, ethanolamine and glycerol-related metabolites likely become available for use.

### Study limitations

One notable limitation of this study that needs to be addressed in future work is that we did not determine the mechanism(s) underlying the observed differences between ticks infected with *B. mayonii* as compared with *B. burgdorferi*. The more substantial decrease in the abundance of purine-related compounds, amino acids, carbohydrates, and fatty acids throughout the blood meal of *B. mayonii*-infected ticks compared to *B. burgdorferi*-infected ticks may suggest that *B. mayonii* places a greater demand for these molecules contained in the blood within the tick vector than *B. burgdorferi*. Alternative explanations could be that the number of spirochetes present within the infected ticks typically is higher for *B. mayonii* than for *B. burgdorferi*, or some as yet unknown aspect of the physiology of *B. mayonii* differing from *B. burgdorferi*. Graphical interpretation of results from the PCR conducted to confirm infection status of individual ticks are suggestive of higher spirochete numbers within ticks infected with *B. mayonii*, as compared with *B. burgdorferi*, but this is not conclusive as the assay was not specifically designed to quantify the amount of spirochete genetic material in the ticks. Another limitation of the study is that we used a single strain of each *Borrelia* species. Examination of additional spirochete strains is required to more confidently generalize our finding for the examined *Borrelia* species. Another important aspect to consider in future studies is whether the observed differences between ticks infected with *B. mayonii* as compared with *B. burgdorferi* actually result in reduced fitness of infected blood fed ticks, for example manifesting as reduced likelihood of *B. mayonii*-infected nymphs to molt to the adult stage or reduced size of female ticks.

## Conclusions

We present the first account of a metabolomic profile of ticks infected with Lyme disease spirochetes, *B. burgdorferi* and *B. mayonii*, which provides insight into the strategies these important human pathogens may employ to adapt to and modify the environment within the tick vector in order to establish infection and continue the transmission cycle. Analogous pathways involved in infection in other pathogenic bacteria, especially enteropathogenic bacteria, may imply that these metabolites play similar roles during *Borrelia* growth and transmission of *Borrelia* by infected ticks. Although the specific roles and significance of these metabolic pathways may be established in other organisms, their functions are not yet fully understood within arthropod vectors. Therefore, the discussions and conclusions made herein remain putative, however, these data do suggest that there are differences in the effects of these two species of Lyme disease spirochetes on the tick metabolome. Future studies repeated over longer time periods could provide insight into aspects of post drop-off metabolism and their potential influence on differences related to transstadial survival and potential transovarial transmission of *Borrelia,* and differences related to subsequent transmission efficacy of the spirochetes.

## Additional Information

**How to cite this article:** Hoxmeier, J. C. *et al*. Metabolomics of the tick-Borrelia interaction during the nymphal tick blood meal. *Sci. Rep.* **7**, 44394; doi: 10.1038/srep44394 (2017).

**Publisher's note:** Springer Nature remains neutral with regard to jurisdictional claims in published maps and institutional affiliations.

## Supplementary Material

Supplementary Figures

## Figures and Tables

**Table 1:** Table of significant features for uninfected (Control) *Ixodes scapularis* nymphs versus nymphs infected with the Lyme disease spirochetes *Borrelia burgdorferi* (Bb) or *Borrelia mayonii* (Bm).

Annotation				p-value			Prefeed-Fold Change		Day 1-Fold Change		Day 2-Fold Change		Day 3-Fold Change		Day 4-Fold Change	
ID	Functional Class	KEGG ID	MSI Conf.	trt	day	trt:day	Control /Bb	Control /Bm	Control /Bb	Control /Bm	Control /Bb	Control /Bm	Control/Bb	Control/Bm	Control/Bb	Control/Bm
Adenosine, alpha- (4TMS) MP	Nucleic Acid	C00212	2	0.0071	0.0014	0.0656	2.5	3.6	1.2	0.9	5.8	7.7	1.5	1.6	0.7	2.6
Adenosine-5-monophosphate (5TMS)	Nucleic Acid	C00020	2	0.0219	<0.0001	0.1554	0.8	0.8	1.3	0.9	0.8	0.8	1.0	1.7	1.0	3.2
Alanine (2TMS)	Amino Acids	C00041	2	0.0048	<0.0001	0.0193	1.1	1.0	1.4	0.5	2.3	1.9	1.3	1.1	1.1	2.3
Allantoin (4TMS)	Ureide	C02350	2	0.1195	0.0001	0.5881	1.0	1.0	1.4	0.7	2.2	1.8	1.3	1.5	0.9	2.0
Benzoic acid, (1TMS)	Co-factor	C00180	1	0.0027	0.0003	0.0041	1.6	0.5	3.6	1.0	1.2	2.0	0.9	0.7	1.5	3.7
Campesterol (1TMS)	Lipid	C01789	2	0.0281	<0.0001	0.0017	0.8	0.8	1.2	1.3	0.9	1.5	0.7	0.6	0.8	2.8
Carbodiimide (2TMS)	Xenobiotic	C01566	2	0.0005	0.0002	0.0106	2.1	1.8	0.3	1.7	0.7	3.7	1.5	1.5	1.2	2.2
Cholesterol (1TMS)	Lipid	C00187	2	0.0004	<0.0001	0.0022	2.2	1.4	2.1	1.2	1.7	2.3	0.8	1.0	1.0	2.6
Cystathionine (4TMS)	Amino Acids	C02291	2	0.0010	<0.0001	0.0013	0.9	0.8	0.7	0.9	0.9	1.7	1.4	1.2	1.2	7.2
Dodecanoic acid (1TMS)	Lipid	C02679	2	<0.0001	0.0003	0.0001	1.8	1.0	2.3	1.3	1.3	1.9	1.2	1.2	1.1	3.2
Ethanolamine (3TMS)	Amine	C00189	2	0.0023	<0.0001	0.0146	1.6	1.3	1.4	1.8	1.5	1.6	1.6	2.5	1.0	5.7
Ethanolaminephosphate (4TMS)	Lipid	C00346	2	0.0083	<0.0001	0.0476	1.7	0.8	1.5	0.8	2.6	2.1	1.2	1.2	1.0	4.2
Galactose (1MEOX) (5TMS) BP.1	Carbohydrates	C00124	2	0.0265	0.0001	0.0305	1.2	1.6	1.6	1.0	1.6	1.5	1.6	1.0	1.7	11.2
Gentiobiose (1MEOX) (8TMS) BP	Carbohydrates (Xeno)	C08240	2	0.0080	0.0001	0.0155	1.1	0.9	1.2	1.5	4.7	5.1	6.5	3.1	0.9	2.9
Glutamic acid (3TMS)	Amino Acids	C00025	2	<0.0001	<0.0001	0.0001	1.5	0.7	1.1	0.3	2.1	1.7	1.2	1.3	1.0	2.4
Glutamine, DL- (3TMS)	Amino Acids	C00064	2	0.1856	<0.0001	0.0249	4.7	0.9	1.8	0.7	2.4	1.8	3.0	1.0	0.6	1.7
Glycerol (3TMS)	Carbohydrates	C00116	2	0.0004	<0.0001	0.0066	1.5	1.5	1.2	1.1	1.7	2.1	1.4	1.3	1.4	3.0
Glycerol-3-phosphate (4TMS)	Lipid	C00093	2	0.0004	<0.0001	0.0025	1.7	0.9	4.2	1.9	3.0	2.6	1.2	1.3	0.8	2.8
Glycine (3TMS)	Amino Acids	C00037	2	<0.0001	<0.0001	0.0009	1.6	1.4	1.4	0.5	2.3	2.0	1.4	1.1	1.2	2.3
Guanine (3TMS)	Nucleic Acid	C00242	2	0.0214	0.0001	0.0797	2.1	1.4	2.1	0.8	2.9	5.0	1.4	2.3	0.6	3.0
Guanosine (5TMS)	Nucleic Acid	C00387	2	0.0025	0.0003	0.0856	4.2	4.2	1.8	0.7	5.2	7.3	1.6	2.0	0.7	3.2
Hexadecanoic acid (1TMS)	Lipid	C00249	2	<0.0001	<0.0001	<0.0001	1.5	0.3	1.0	0.3	1.1	0.3	0.9	0.4	1.0	1.1
Hypoxanthine (2TMS)	Nucleic Acid	C00262	2	0.1108	0.0002	0.5390	1.0	1.0	1.5	0.8	8.4	8.4	1.1	1.2	1.0	2.4
Inosine (4TMS)	Nucleic Acid	C00294	2	0.0595	<0.0001	0.3658	74.9	4.5	39.1	29.3	0.0	0.0	0.5	1.6	1.8	17.2
Isoleucine (2TMS)	Amino Acids	C00407	2	0.0045	<0.0001	0.0211	1.5	0.4	2.1	0.2	3.4	2.6	2.1	1.2	1.2	3.9
Leucine (1TMS)	Amino Acids	C00123	2	0.0017	<0.0001	0.0134	1.5	1.1	1.9	0.5	3.7	4.2	1.8	1.1	1.3	4.1
Lysine (4TMS)	Amino Acids	C00047	2	0.0009	<0.0001	0.0011	1.6	1.5	0.9	0.4	2.4	1.5	1.5	1.5	1.0	3.2
Maltose (1MEOX) (8TMS) MP	Carbohydrates	C00208	2	0.0036	<0.0001	0.0071	1.5	1.9	1.3	1.6	4.8	6.7	5.5	3.3	0.8	2.9
myo-Inositol-1-phosphate (7TMS)	Co-factor	C01177	2	0.0016	<0.0001	0.0184	1.5	1.5	1.1	0.8	2.6	2.1	1.0	1.1	1.0	2.3
NA114002 (classified unknown)	unknown	unknown	2	0.0005	<0.0001	0.0012	1.3	0.9	1.1	1.0	1.2	1.2	1.1	1.0	1.0	1.1
Octadecenoic acid, 9-(Z)- (1TMS)	Lipid	C00712	2	<0.0001	<0.0001	0.0017	1.4	0.7	1.9	1.4	1.7	2.7	1.3	1.2	1.2	3.6
Ornithine (4TMS)	Amino Acids	C00077	2	0.0045	<0.0001	0.0072	2.5	3.2	1.5	0.7	1.7	1.5	1.5	1.1	1.0	3.8
Pantothenic acid, D- (3TMS)	Vitamins	C00864	2	<0.0001	<0.0001	0.0002	1.1	0.8	1.6	0.8	3.6	2.9	2.1	1.8	1.1	3.0
Phenylalanine (2TMS)	Amino Acids	C00079	2	0.0003	<0.0001	0.0017	1.8	0.9	1.9	0.4	1.9	2.5	2.2	1.4	1.4	4.7
Phosphoric acid (3TMS)	Co-factor	C00009	2	0.0004	<0.0001	0.0015	1.0	1.1	0.9	1.3	1.5	1.7	1.0	1.0	1.0	2.0
Phosphoric acid monomethyl ester (2TMS)	Xenobiotic	C19998	2	0.0042	<0.0001	0.0095	1.0	1.2	1.0	0.7	4.0	2.4	0.6	1.2	0.9	3.3
Proline (1TMS)	Amino Acids	C00148	2	<0.0001	<0.0001	<0.0001	1.1	1.0	1.0	1.0	2.3	1.6	1.1	0.8	1.1	4.7
Pyroglutamic acid (2TMS)	Amino Acids	C01879	2	<0.0001	<0.0001	0.0006	2.6	1.2	1.4	0.8	2.0	1.7	1.3	1.6	0.9	2.8
Serine (3TMS)	Amino Acids	C00065	2	0.0012	<0.0001	0.0052	1.8	1.0	1.1	0.5	1.9	1.9	1.7	1.4	1.0	3.3
Sitosterol, beta- (1TMS)	Lipid	C01753	2	0.0874	<0.0001	0.0078	0.9	1.4	1.0	1.5	0.5	1.5	0.5	0.4	0.8	3.3
Sucrose (8TMS)	Carbohydrates	C00089	2	0.0010	0.2407	0.0043	2.2	1.0	1.1	0.1	1.2	0.1	1.7	1.2	1.0	1.4
Threonine (3TMS)	Amino Acids	C00188	2	0.0024	<0.0001	0.0236	1.5	1.1	1.2	0.5	2.0	2.4	1.9	1.1	1.3	3.2
Tryptophan (3TMS)	Amino Acids	C00078	2	0.1798	<0.0001	0.0103	1.3	0.5	1.0	0.6	1.5	0.8	1.0	0.9	0.9	1.6
Tyrosine (3TMS)	Amino Acids	C00082	2	0.0028	<0.0001	0.0136	1.2	1.2	1.6	0.5	1.6	1.6	1.5	1.4	1.0	3.3
Uracil (2TMS)	Nucleic Acid	C00106	2	0.0021	<0.0001	0.0471	1.2	0.8	0.5	0.5	2.4	2.0	1.1	1.1	1.2	2.1
Urea (2TMS)	Amino Acids	C00086	2	0.0045	<0.0001	0.0366	1.1	0.5	2.3	0.6	2.9	2.9	1.0	1.1	1.3	2.8
Valine (2TMS)	Amino Acids	C00183	2	<0.0001	<0.0001	0.0002	1.4	0.7	1.2	0.4	3.1	2.3	2.1	1.4	1.1	3.8
Xanthine (3TMS)	Nucleic Acid	C00385	2	0.0001	<0.0001	0.0001	1.1	0.8	1.0	0.6	6.5	4.4	0.8	1.2	0.7	0.7
Xanthosine (5TMS)	Nucleic Acid	C01762	2	0.2085	<0.0001	0.0340	2.1	0.9	1.3	0.2	7.0	1.3	1.4	1.4	0.4	1.6

A. KEGG: Kyoto Encyclopedia of Genes and Genomes; B. MSI Conf. Metabolomics Standards Initiative confidence of annotation; C. p-value significance by ANOVA analysis of treatment (trt: *Borrelia* infection status), time, and treatment:time (trt:time). D. Fold change determined by dividing relative abundance of selected metabolite in control ticks by relative abundance of metabolite in the *Borrelia* infected groups.
