# Supplementary material for: Metabolomics of the tick-Borrelia interaction during the nymphal tick blood meal
Source: Sci Rep. 2017 Mar 13;7:44394. doi: 10.1038/srep44394 (PMC5347386; doi:10.1038/srep44394)

Metabolomics of the tick-*Borrelia* interaction during the nymphal tick blood meal

J. Charles Hoxmeier[1]‡, Amy C. Fleshman[1]‡*, Corey D. Broeckling[2], Jessica E. Prenni[2], Marc C. Dolan[1], Kenneth L. Gage[1], and Lars Eisen[1]

Supplementary Information

Supplementary Figure 1: Purine Pathway Trends Flux over time of statistically significant (ANOVA p≤0.05) metabolites involved in purine metabolic pathway in uninfected (control) *Ixodes scapularis* nymphs versus nymphs infected with the Lyme disease spirochetes *Borrelia burgdorferi* or *Borrelia mayonii*. TMS:trimethylsilyl derivative from GC-MS analysis.

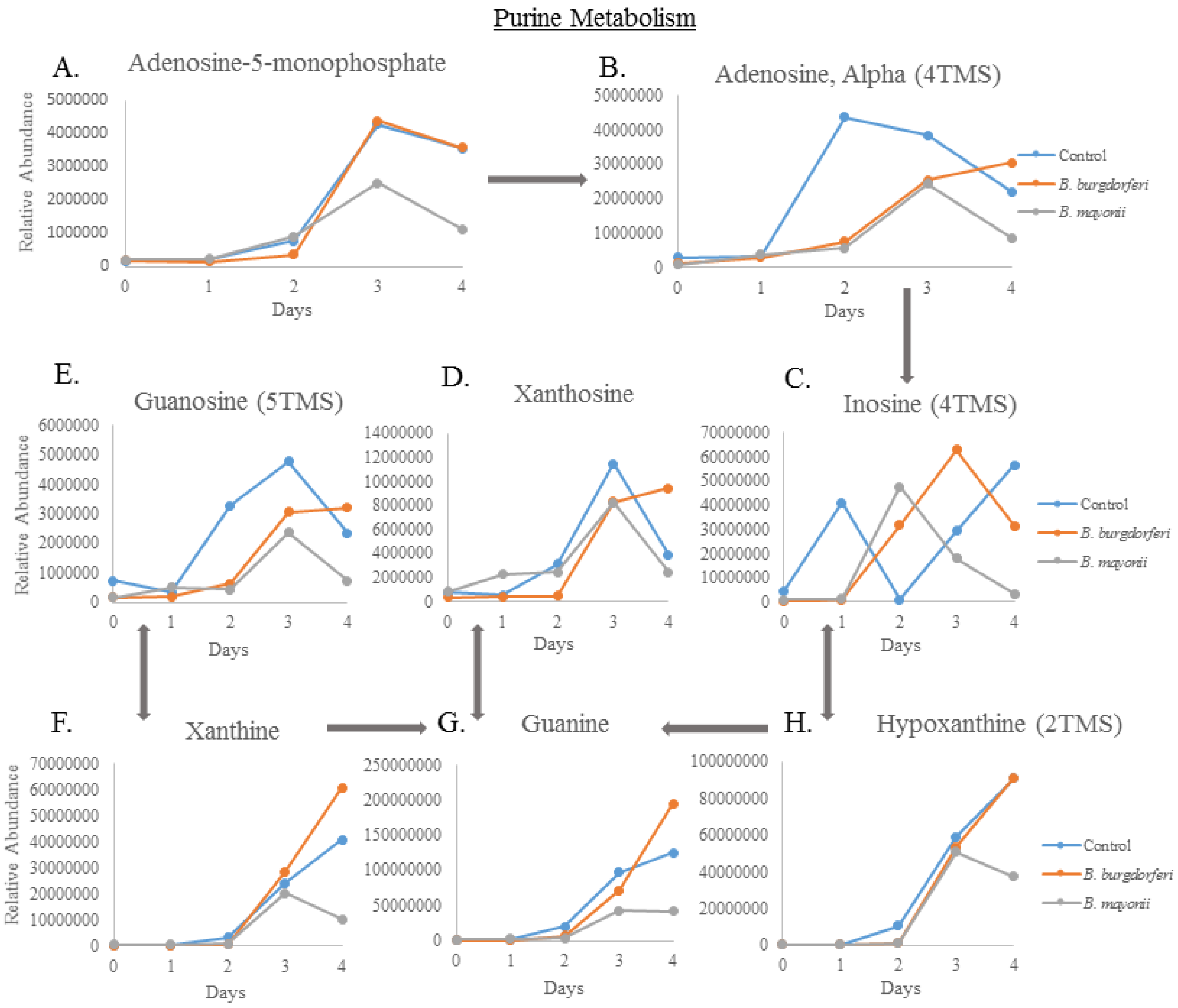

Supplementary Figure 2: Amino Acid Trends Accumulation over time of statistically significant (ANOVA $p \leq 0.05$) amino acids in uninfected (control) *Ixodes scapularis* nymphs versus nymphs infected with the Lyme disease spirochetes *Borrelia burgdorferi* or *Borrelia mayonii*.

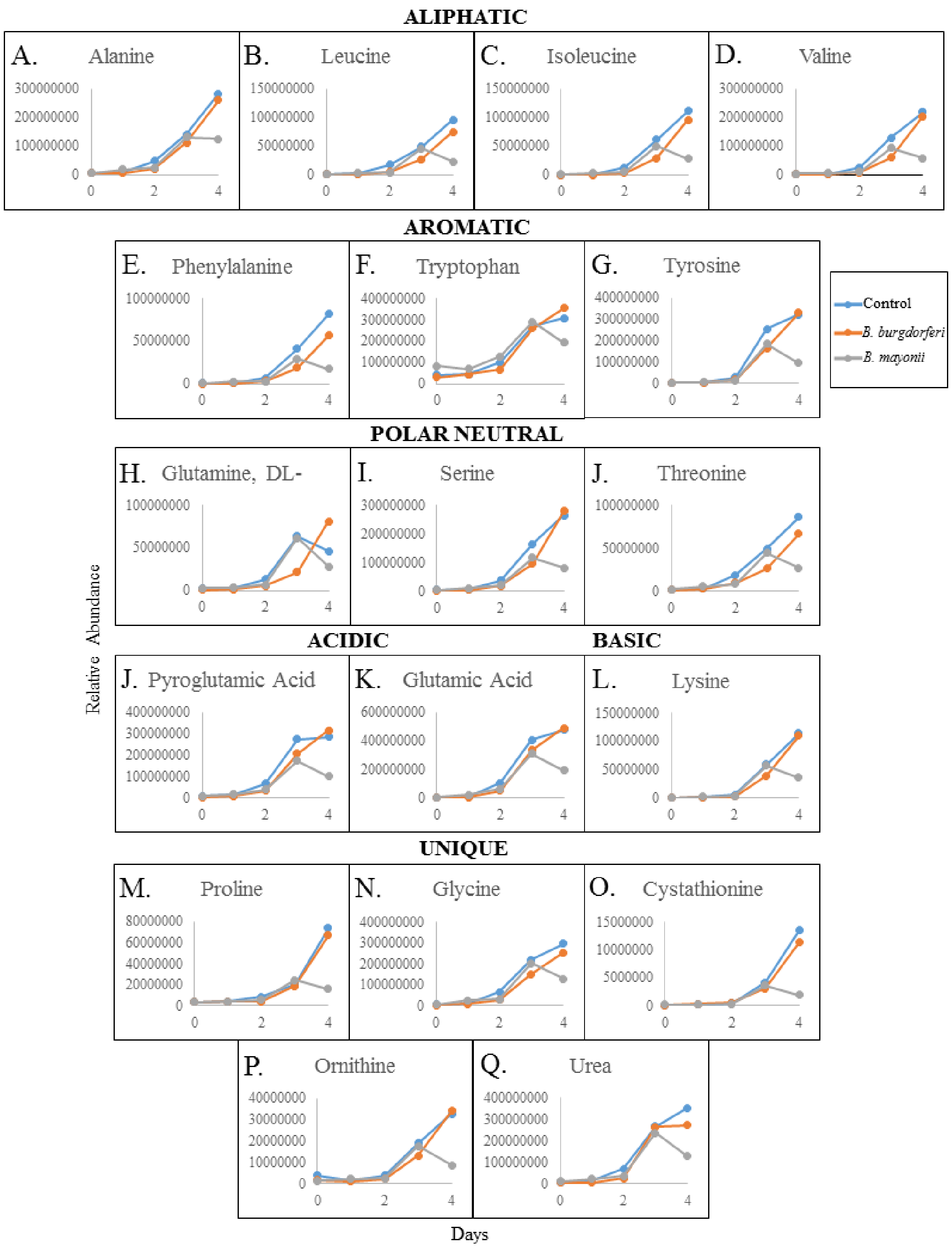

Supplementary Figure 3 Part 1: Amino Acids Box and Whisker Plots (1 of 3) Accumulation over time of statistically significant (ANOVA p≤0.05) amino acids in uninfected (control) *Ixodes scapularis* nymphs versus nymphs infected with the Lyme disease spirochetes *Borrelia burgdorferi* or *Borrelia mayonii*.

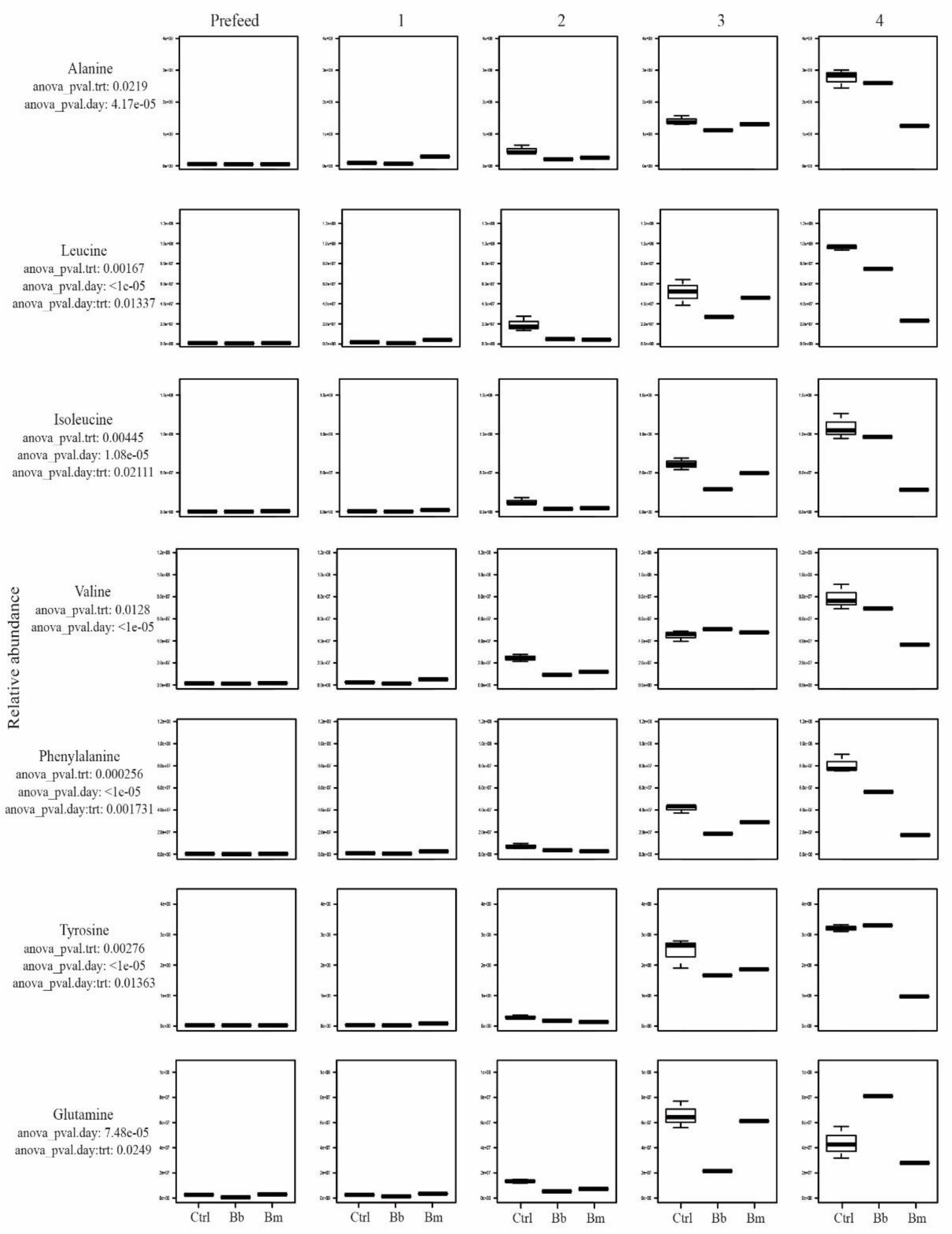

Supplementary Figure 3 Part 2: Amino Acids Box and Whisker Plots (2 of 3) Accumulation over time of statistically significant (ANOVA p≤0.05) amino acids in uninfected (control) *Ixodes scapularis* nymphs versus nymphs infected with the Lyme disease spirochetes *Borrelia burgdorferi* or *Borrelia mayonii*.

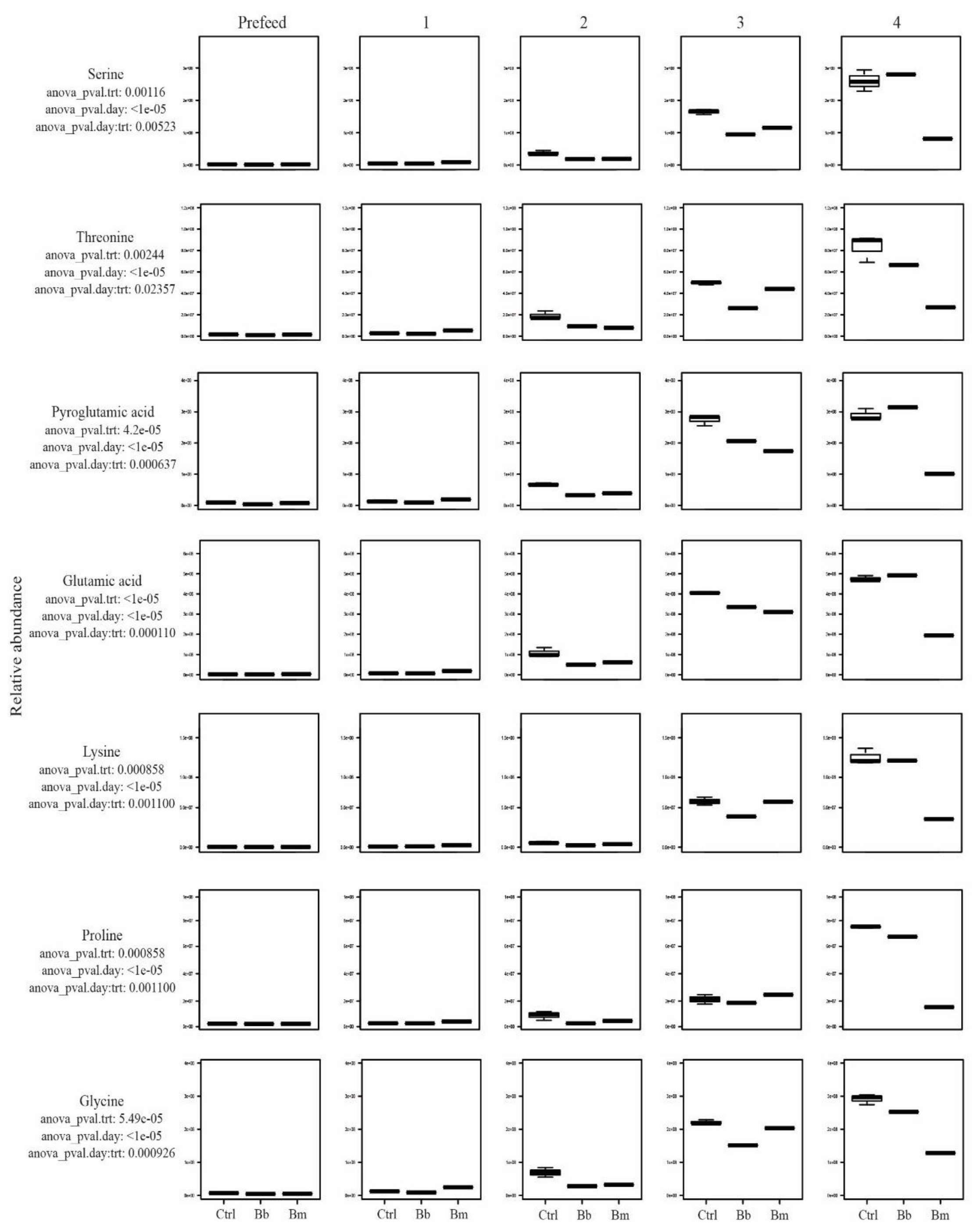

Supplementary Figure 3 Part 3: Amino Acids Box and Whisker Plots (3 of 3) Accumulation over time of statistically significant (ANOVA p≤0.05) amino acids in uninfected (control) *Ixodes scapularis* nymphs versus nymphs infected with the Lyme disease spirochetes *Borrelia burgdorferi* or *Borrelia mayonii*.

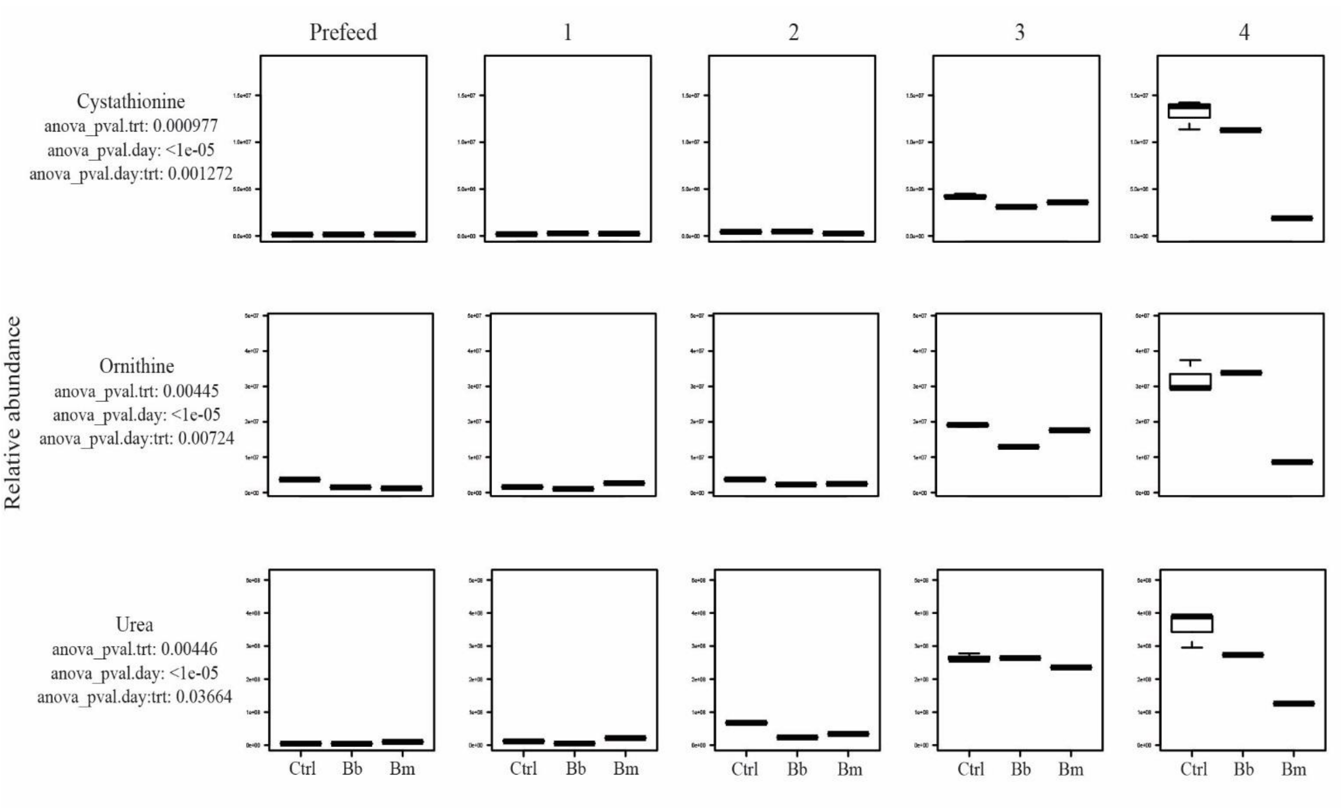

Supplementary Figure 4: Carbohydrate Trends Accumulation over time of statistically significant (ANOVA $p \leq 0.05$) carbohydrates in uninfected (control) *Ixodes scapularis* nymphs versus nymphs infected with the Lyme disease spirochetes *Borrelia burgdorferi* or *Borrelia mayonii*.

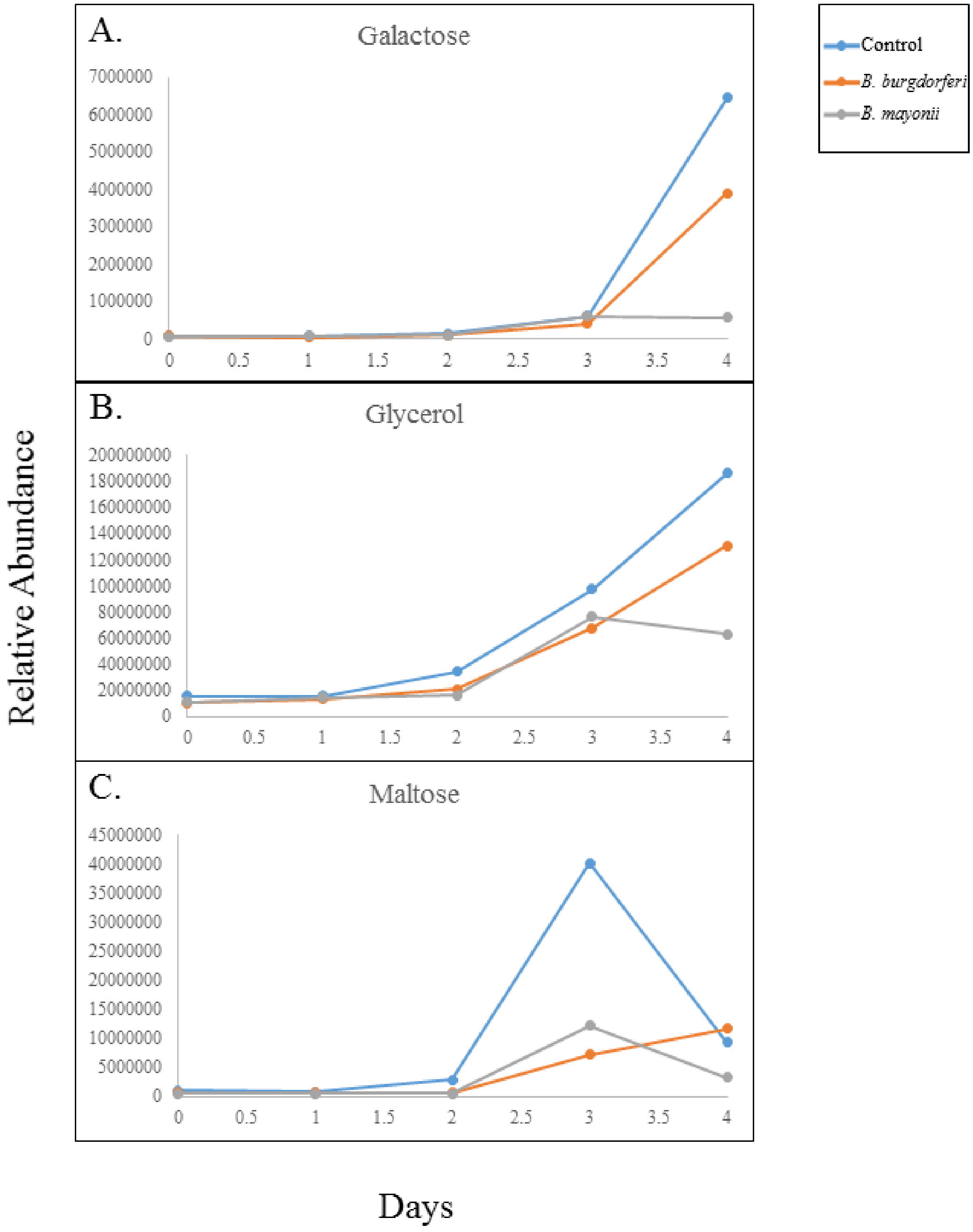

Supplementary Figure 5: Fatty Acid Trends Accumulation over time of statistically significant (ANOVA $p \leq 0.05$) lipids in uninfected (control) *Ixodes scapularis* nymphs versus nymphs infected with the Lyme disease spirochetes *Borrelia burgdorferi* or *Borrelia mayonii*.

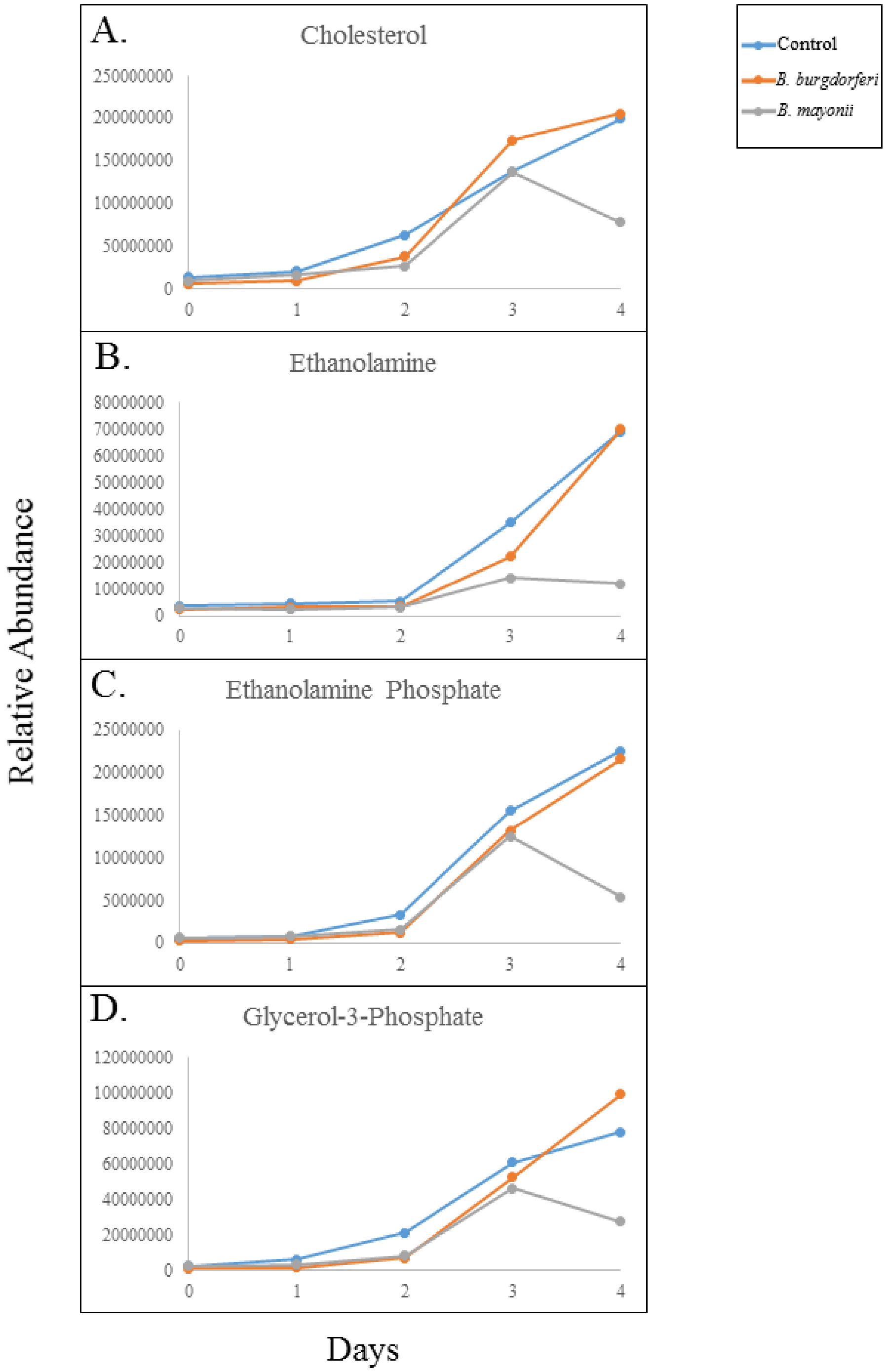

Supplementary Figure 6: Histogram of Cluster CVs of QC Samples Histogram of the coefficients of variation (CV) for all compounds detected, showing that the majority of ion signals have CV values below 20% in the QC (quality control) samples. QC samples were pooled from all samples in dataset.

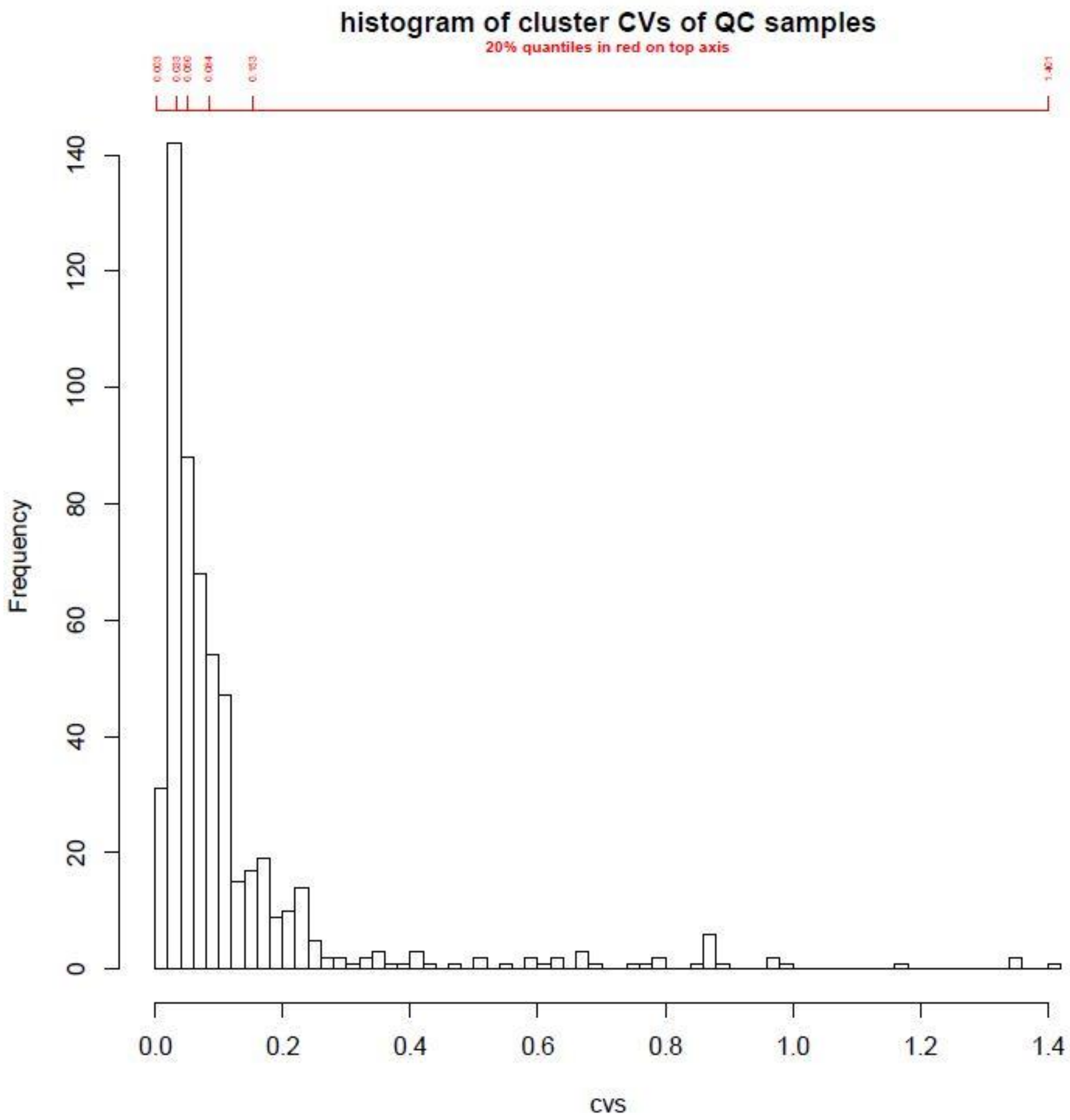

Supplement: Supplementary Figures [file srep44394-s1.pdf]
